# An Insight Into the Distribution of Allele Frequency of ABO and Rh (D) Blood Grouping System Among Blood Donors in a Tertiary Care Hospital in Chengalpattu District of South India

**DOI:** 10.7759/cureus.24207

**Published:** 2022-04-17

**Authors:** Karthik Sigamani, Shiva Prasad Gajulapalli

**Affiliations:** 1 Department of Pathology, Karpaga Vinayaga Institute of Medical Sciences and Research Centre, Chengalpattu, IND

**Keywords:** prevalence, abo grouping, d antigen, genotype, phenotype

## Abstract

Introduction

The distribution of ABO and Rh (D) blood groups and their allele frequencies vary from one population to another worldwide. The objective of the study is to estimate the distribution of ABO & Rh (D) blood groups among all the blood donors in a tertiary care hospital in Chengalpattu district of Tamilnadu in South India and to determine their allele frequencies.

Methods

This was a retrospective observational study carried out in the blood bank of Karpaga Vinayaga Institute of Medical Sciences and Research Centre from January 2015 to December 2021. ABO and Rh (D) blood grouping of all the blood donors were carried out by tube agglutination method. Allele frequency of the blood group genes was calculated based on Hardy-Weinberg equilibrium.

Results

Out of total a of 7598 blood donors, 7576 (99.71%) were males and 22 (0.29%) were females. The most common blood group was O positive (37.67%) while AB negative (0.18%) was the least common blood group. The phenotypic frequency of blood group O (39.17%) was the highest and that of blood group AB (7.88%) was the least. A majority (95.96%) of the blood donors were Rh (D) positive. The allele frequencies of ABO and Rh (D) blood groups were 0.1628 for I^A^, 0.2177 for I^B^, 0.6259 for I^O^, 0.7991 for I^D,^ and 0.2009 for I^d^.

Conclusions

The distribution of the two major blood group systems namely ABO and Rh (D) systems show considerable heterogeneity in different populations of the world. Information about allele frequencies of blood groups among different populations worldwide will help in framing policy decisions to face future challenges in healthcare services.

## Introduction

Blood groups of individuals are genetically determined and are classified based on the presence of specific antigens on the surface of red blood cells (RBCs). These blood group antigens follow the mendelian mode of inheritance and have stable characteristics [1]. These antigens appear in the early fetal period and persist throughout the life unchanged [2]. Among the 30 different blood group systems that have been described by the International Society of Blood Transfusion, ABO and Rh systems are considered the most significant major blood group systems. These two major blood group systems play an important role in blood transfusion and organ transplantation [3]. Australian scientist Karl Landsteiner was awarded the Nobel Prize for his discovery of the ABO system in 1930 while the Rh system was discovered in 1940 by Karl Landsteiner and Weiner. The antigens that determine the ABO system are A & B antigens while the original significant and most immunogenic Rh antigen was referred to as D and the related Rh antigens are C, E, c & e [2]. The ABO system antigens, A and B are strongly immunogenic in nature and are located on the surface of RBCs while the antibodies, anti-A, and anti-B are present naturally in the plasma of individuals whose RBCs lack the corresponding antigen [3]. In 1940, Levine and Stetson linked hemolytic disease of newborns to the Rh blood group system (D antigen) [4]. The genes for ABO and Rh antigens are located on chromosome nine (9q) and chromosome one (1p) respectively [5]. Most of these blood group genes are expressed in a co-dominant manner [1]. The ABO system is characterised by three alleles of the ABO genes namely I^A^, I^B,^ and I^O^ with six possible genotypes among the population namely I^A^I^A^, I^A^I^O^, I^B^I^B^ , I^B^I^O^ , I^A^I^B^ and I^O^I^O^ [4]. The resultant four ABO phenotypes are blood group A (I^A^I^A^ & I^A^I^O^), blood group B (I^B^I^B^ & I^B^I^O^), blood group AB (I^A^I^B^), and blood group O (I^O^I^O^ ). The genotypes of Rh (D) positive individuals are I^D^I^D^ or I^D^I^d^ while the genotype of Rh (D) negative individuals is I^d^I^d^ [4].

The distribution of ABO and Rh (D) blood groups varies from one population to another worldwide and also within different regions of a country. Information regarding the distribution of ABO and Rh (D) blood groups and their allele frequencies among different populations of the world is helpful for effective inventory management in blood banks [2] and also useful in resolving medico-legal issues like disputed paternity, population genetic studies and researching population migration patterns [3]. ABO system is also known to be associated with certain diseases like urinary tract infection, duodenal ulcers, gastric carcinoma, and diabetes mellitus [6]. Studies have been conducted in different parts of India to determine the ABO and Rh (D) blood groups' allele frequency distribution among the local population. However, there are no similar studies carried out in the Chengalpattu district of Tamilnadu in South India. Hence, this study is intended to estimate the distribution of ABO & Rh (D) blood groups among all the blood donors in a tertiary care hospital in Chengalpattu district of Tamilnadu in South India and to determine the allele frequency distribution of the ABO and Rh (D) blood group systems.

## Materials and methods

Setting

This was a retrospective observational study carried out in the blood bank of Karpaga Vinayaga Institute of Medical Sciences and Research Centre, Chengalpattu district, Tamilnadu. The study period was seven years from January 2015 to December 2021.

Inclusion criteria

The inclusion criteria for the study was defined as: 

a) All blood donors are more than 18 years and less than 60 years of age.

b) All blood donors without any chronic medical illness.

c) All blood donors with a bodyweight of more than 45 kg.

d) All blood donors with hemoglobin of more than 12.5 g/dl.

Exclusion criteria

The exclusion criteria was defined as:

a) Voluntary blood donors having a frequency of blood donation of fewer than three months.

b) Blood donors on medications.

c) Blood Donors with a history of recent surgery.

Sample size

The purposive sampling technique was used for the selection of desired samples according to the inclusion criteria.

Protocol

The study was approved by the Karpaga Vinayaga Institute of Medical Sciences and Research Centre- Institutional Ethics Committee (Human Studies). Informed consent was obtained from all blood donors during blood donation. After blood donation, ABO grouping and Rh (D) typing of the donor blood samples were carried out using the tube agglutination method. In ABO blood grouping, both forward grouping and reverse grouping were performed. Forward grouping was done using commercially available monoclonal antisera manufactured by Tulip diagnostics namely anti-A and anti-B. All “O” blood group samples were tested with anti-H lectin to exclude the Bombay blood group. Reverse grouping was performed by using pooled A cells, B cells, and O cells prepared in the blood bank. The final blood group was based on correlating identical forward and reverse grouping. For Rh (D) typing, commercially available antisera, anti-D (anti-IgM & blend of anti-IgM /anti-IgG) were used. All Rh (D) negative blood samples were confirmed with an anti-globulin test (Du test). All weak D blood samples detected by the Du test were considered Rh (D) positive.

Statistical analysis

All the donor ABO and Rh (D) blood groups were tabulated in Microsoft Excel and analyzed. Descriptive statistical measures like frequency and percentage were calculated.

Allele frequency of the ABO and Rh (D) blood group genes was calculated based on Hardy-Weinberg equilibrium using the following equations:

p =1− √(B + O); q =1− √(A + O); r = √O; E = 1‑e; e =√dd

where p, q, r, E, and e represent the allele frequencies of the genes for A, B, O, Rh (D) positive and Rh (D) negative respectively. A, B, O, and dd represent the phenotype frequencies of blood groups A, B, O, and Rh (D) negative respectively [4].

## Results

During the seven years study period from January 2015 to December 2021, a total of 7598 donor blood samples were tested for ABO grouping and Rh (D) typing, out of which 7576 (99.71%) were from male donors and 22 (0.29%) were from female donors. The most common blood group in this study was O positive accounting for 37.67% while AB negative was the least common blood group accounting for 0.18% (Table 1).

**Table 1:** Distribution of ABO & Rh (D) blood groups among Blood Donors

S. No	Blood group	Total Blood Donors	Percentage
1	O positive	2862	37.67%
2	B positive	2257	29.71%
3	A positive	1587	20.89%
4	AB positive	585	7.7%
5	O negative	114	1.5%
6	B negative	92	1.21%
7	A negative	87	1.15%
8	AB negative	14	0.18%
	Total	7598	100%

Among males, the most common blood group was O positive (37.7%) while B positive (36.36%) was the commonest blood group among females (Table 2).

**Table 2:** Genderwise distribution of ABO & Rh (D) blood groups

S. No	Blood group	Male Blood Donors		Female Blood Donors	
		Total	Percentage	Total	Percentage
1	O positive	2856	37.7%	6	27.27%
2	B positive	2249	29.69%	8	36.36%
3	A positive	1583	20.89%	4	18.19%
4	AB positive	583	7.7%	2	9.09%
5	O negative	112	1.48%	2	9.09%
6	B negative	92	1.21%	0	0
7	A negative	87	1.15%	0	0
8	AB negative	14	0.18%	0	0
	Total	7576	100%	22	100%

In this study, the phenotypic frequency and prevalence rate of blood group O (39.17%) was the highest and the prevalence rate of blood group AB (7.88%) was the least (Table 3). The majority of the blood donors were Rh (D) positive with a prevalence rate of 95.96% (Table 3).

**Table 3:** Prevalence rate of ABO & Rh (D) blood groups among Blood Donors

Blood group system	Phenotype	Total Blood Donors	Blood group Frequency	Prevalence rate	
					ABO system	A	1674	0.2203	22.03%	
B	2349	0.3092	30.92%		
O	2976	0.3917	39.17%		
AB	599	0.0788	7.88%		
Rh (D) system	Rh (D) positive	7291	0.9596	95.96%	
	Rh (D) negative	307	0.0404	4.04%	

The allele frequencies of ABO and Rh (D) blood group genes calculated based on Hardy-Weinberg equilibrium were 0.1628 for I^A^, 0.2177 for I^B^, 0.6259 for I^O^, 0.7991 for I^D,^ and 0.2009 for I^d^ (Table 4).

**Table 4:** Distribution of allele frequency of ABO & Rh (D) blood groups among Blood Donors

Blood group system	Blood group allele	Allele Frequency
ABO system	I^A^	0.1628
	I^B^	0.2177
	I^O^	0.6259
Rh (D) system	I^D^	0.7991
	I^d^	0.2009

## Discussion

Blood transfusion is an integral part and life-saving measure of the modern healthcare system. Knowledge of the distribution of blood groups among different populations worldwide is essential for the recruitment of blood donors. The majority of studies that were conducted within India reported more number of male blood donors compared to female blood donors [4] similar to the present study where male blood donors accounted for 99.71% of the total blood donors. However, studies conducted in other countries reported a slightly higher proportion of female blood donors compared to Indian studies. Iyiola et al. [7] from Nigeria reported 47.1% of female blood donors and Woldu et al. [8] from Ethiopia reported 17.9% of female blood donors.

The phenotype and genotype frequencies of ABO and Rh (D) blood groups show considerable variation across different population groups, races, and geographical boundaries [4]. The distribution of ABO and Rh (D) blood groups in the present study conducted in the Chengalpattu district of South India was compared with several other studies conducted in different regions within and outside India.

In the present study, blood group O was the commonest followed by B, A, and AB in concordance with other South Indian studies by Das et al. from Vellore [9], Periyavan et al. from Bangalore [10], and Suresh et al. from Tirupati [1]. In the studies conducted by Kumar S et al. [4] and Mohroo et al. [11] from North India, blood group B showed the highest frequency while blood group AB showed the least frequency. Studies by Kumar M et al. [12] and Raja et al. [13] from Western India showed results similar to the North Indian studies. The phenotypic frequencies of blood groups showed intra-regional variations in the studies conducted in Central and Eastern India. In Central India, Mehta et al. [14] reported the highest frequency of blood group B in Madhya Pradesh while Badge et al. [15] reported the highest frequency of blood group O in Chattisgarh. In Eastern India, Singh et al. [6] reported the highest frequency of blood group B in Ranchi while Ipsita et al. [16] reported the highest frequency of blood group O in Durgapur. In a study by Giri et al. [17] from Maharashtra located in Western and Central India, blood group B showed the highest frequency closely followed by blood group O (Table 5).

**Table 5:** Comparison of Prevalence of ABO & Rh (D) blood groups in different regions of India

Region	Place of study	Author	A group (%)	B group (%)	O group (%)	AB group (%)	Rh (D) positive (%)	Rh (D) negative (%)
South India	Chengalpattu district, Tamilnadu	Present study	22.03	30.92	39.17	7.88	95.96	4.04
	Vellore, Tamilnadu	Das et al. [9]	18.85	32.69	38.75	5.27	94.5	5.47
	Bangalore, Karnataka	Periyavan et al. [10]	23.85	29.95	39.81	6.37	94.20	5.7
	Tirupati, Andhra Pradesh	Suresh et al. [1]	20.0%	32.2%	41.7%	6.1%	92.8%	7.2%
North India	Srinagar, Uttarakhand	Kumar S et al. [4]	30.39	31.68	26.24	11.70	93.51	6.49
	Delhi	Mohroo RN et al. [11]	23.98	35.40	30.96	9.65	95.63	4.37
Central India	Madhya Pradesh	Mehta et al. [14]	25.63	39.25	28.63	6.50	94.88	5.12
	Chattisgarh	Badge et al. [15]	24.95	30.44	31.09	13.52	99.43	0.57
Eastern India	Ranchi, Jharkhand	Singh et al. [6]	22.09	35.15	34.73	8.03	96.46	3.54
	Durgapur	Ipsita et al. [16]	23.9	33.6	34.8	7.7	94.7	5.3
Western India	Rajasthan	Kumar M et al. [12]	15.46	39.95	35.68	8.80	91.17	8.82
	Gujarat	Raja et al. [13]	24.35	34.43	32.26	8.94	95.12	4.87
Western & Central India	Maharashtra	Giri et al. [17]	28.38	31.89	30.99	8.72	95.36	4.64

In studies conducted outside India, blood group O was the commonest group in Iran, Saudi Arabia, Nigeria, Ethiopia, the USA, and Britain except in Bangladesh and Pakistan where blood group B was the commonest one. The majority of the population worldwide was Rh (D) positive similar to the present study. There was a slightly higher proportion of Rh (D) negative individuals in the USA (15%) and Britain (17%) compared to other populations of the world (Table 6).

**Table 6:** Comparison of Prevalence of ABO & Rh (D) blood groups in different world populations

Place of study	A group (%)	B group (%)	O group (%)	AB group (%)	Rh (D) positive (%)	Rh (D) negative (%)
India (Present study)	22.03	30.92	39.17	7.88	95.96	4.04
Bangladesh [4]	26.57	34.15	29.67	9.61	90.82	9.18
Pakistan [5]	27.92	32.40	29.10	10.58	90.13	9.87
Iran [4]	28.48	24.71	40.21	6.60	92.38	7.62
Saudi Arabia [5]	24.0	17.0	52.0	4.0	93.0	7.0
Nigeria [7]	23.1	21.3	52.9	2.70	97.0	3.0
Ethiopia [8]	26.44	21.71	47.04	4.81	94.24	5.76
USA [5]	41.0	9.0	46.0	4.0	85.0	15.0
Britain [5]	42.0	8.0	47.10	3.0	83.0	17.0

The allele frequency of ABO and Rh (D) blood group genes calculated in the present study was compared with other studies conducted within and outside India [18-24]. All the studies showed results concordant with the present study with the highest allele frequency for I^O^ in the ABO system and I^D^ in the Rh (D) system (Table 7). 

**Table 7:** Comparison of distribution of ABO & Rh (D) blood groups allele frequency in different populations of the world

Place of study	Authors	ABO system Allele frequency			Rh (D) system Allele frequency	
		I^A^	I^B^	I^O^	I^D^	I^d^
Within India						
Chengalpattu district, Tamilnadu	Present study	0.1628	0.2177	0.6259	0.7991	0.2009
Srinagar, Uttarakhand	Kumar S et al. [4]	0.2403	0.2475	0.5122	0.7452	0.2548
Multicentric study, India	Agrawal et al. [3]	0.1653	0.2254	0.6093	0.7679	0.2321
Tirupati, Andhra Pradesh	Suresh et al. [1]	0.1398	0.2148	0.6454	0.7321	0.2679
South Gujarat	Raja et al. [13]	0.1844	0.2477	0.5679	0.7794	0.2206
Punjab	Sidhu et al. [18]	0.1710	0.2700	0.5590	0.8360	0.1640
Chattisgarh	Shrivastava et al. [19]	0.1716	0.2512	0.5792	0.8234	0.1766
Outside India						
Lagos, Nigeria	Iyiola et al. [7]	0.14	0.13	0.73	0.97	0.03
Ethiopia	Woldu et al. [8]	0.1714	0.1433	0.6859	0.7599	0.2401
Malaysia	Amin et al. [20]	0.17	0.20	0.63	0.73	0.27
Oman	Allawati et al. [21]	0.15	0.14	0.71	0.71	0.29
Iraq	Al-Ani et al. [22]	0.20	0.14	0.64	0.65	0.35
Mexico (North West)	Canizalez-Roman et al. [23]	0.1676	0.0562	0.7762	0.7855	0.2145
Multan, Pakistan	Zafar et al. [24]	0.2330	0.1714	0.6062	0.9980	0.0900

Limitations

Less number of female blood donors in the present study doesn’t capture the exact prevalence rates of the ABO and Rh (D) blood groups in the general female population. Blood donation by females has to be increased by creating awareness about blood donation and improving the health status of females.

## Conclusions

The distribution of the two major blood group systems namely ABO and Rh (D) systems show considerable heterogeneity in different populations of the world. This could be related to genetic and environmental factors. Worldwide the most common blood group in the ABO system was either O or B blood group with AB being the least common group. In the Rh system, more than 80% of the world population were positive for the D antigen and are Rh-positive with less number of Rh (D) negative individuals. The allele frequencies of the ABO and Rh (D) blood group genes don’t differ significantly from the phenotype frequencies of the ABO and Rh (D) blood group systems.

Though there are several studies carried out worldwide to understand the phenotypic frequencies of ABO and Rh (D) blood groups, only limited data is available regarding the allele frequencies of these major blood group systems. Several such studies on allele frequencies should be carried out worldwide that will be very useful in framing policy decisions to face future challenges in transfusion medicine and healthcare services.

## References

[1] Suresh B, Sreedhar Babu KV, Chandra Mouli P, Arun R, Jothibai DS (2015). Distribution of ABO and rhesus (D) blood group antigens among blood donors at a tertiary care teaching hospital blood bank in south India. J Clin Sci Res.

[2] Bollipogu S, Malothu D, Bajaj NK (2018). Distribution of ABO and Rhesus blood groups in blood donors at blood bank of tertiary care hospital, Mahabubnagar, Telangana. MIJOPAT.

[3] Agrawal A, Tiwari AK, Mehta N, Bhattacharya P, Wankhede R, Tulsiani S, Kamath S (2014). ABO and Rh (D) group distribution and gene frequency; the first multicentric study in India. Asian J Transfus Sci.

[4] Kumar S, Modak PK, Ali SH, Barpanda SK, Gusain VS, Roy R (2018). A retrospective study: ABO and Rh phenotype blood group distribution among blood donors in H.N.B. Base Hospital, Srinagar, Uttarakhand, India. J Family Med Prim Care.

[5] Reddy MK, Kumar RK, Reddy DS (2019). ABO and Rhesus Types of blood group distribution in blood donors in blood bank, Government Medical College and Hospital-Suryapet, Telangana: a tertiary care centre study. Trop J Path Micro.

[6] Singh A, Srivastava RK, Deogharia KS, Singh KK (2016). Distribution of ABO and Rh types in voluntary Blood donors in Jharkhand area as a study conducted by RIMS, Ranchi. J Family Med Prim Care.

[7] Iyiola OA, Igunnugbemi OO, Bello OG (2012). Gene frequencies of ABO and Rh(D) blood group alleles in Lagos, South-West Nigeria. Egypt J Med Hum Genet.

[8] Woldu B, Melku M, Shiferaw E, Biadgo B, Abebe M, Gelaw Y (2022). Phenotype, allele and genotype frequency of ABO and Rhesus D blood groups of blood donors at the North Gondar District Blood Bank, Northwest Ethiopia. J Blood Med.

[9] Das PK, Nair SC, Harris VK, Rose D, Mammen JJ, Bose YN, Sudarsanam A (2001). Distribution of ABO and Rh-D blood groups among blood donors in a tertiary care centre in South India. Trop Doct.

[10] Periyavan S, Sangeetha SK, Marimuthu P, Manjunath BK, Seema DM (2010). Distribution of ABO and Rhesus-D blood groups in and around Bangalore. Asian J Transfus Sci.

[11] Mohroo RN, Hassan MJ, Khan S, Ahmad N, Jetley S (2020). Distribution pattern of ABO and Rh blood group among blood donors at Hospital Blood Bank in Delhi- an initial step to evaluate preparedness to fight an epidemic. IABCR.

[12] Kumar M, Rathore SP (2018). Distribution of blood groups in blood donors in the blood bank, Jhalawar Hospital & Medical College Society, Jhalawar, Rajasthan. IOSR Journal of Dental and Medical Sciences.

[13] Raja KA, Dobariya GH, Unagar CA, Pandya AN, Patel JN, Wadhwani SN (2016). Frequency and distribution of ABO and Rh blood groups among blood donors in tertiary care hospital of South Gujarat. India. Int J Res Med Sci.

[14] Mehta AA, Mehta AA (2016). Frequency distribution of ABO blood group and Rh factor in Bhanpur, Bhopal. Sch Acad J Biosci.

[15] Badge SA, Ovhal AG, Azad K, Meshram AT (2017). Distribution of blood groups in blood donors in the blood bank of Jagdalpur, Bastardistrict, Chattisgarh. Med J DY Patil Univ.

[16] Nag I, Das SS (2012). ABO and Rhesus blood groups in potential blood donors at Durgapur Steel city of the district of Burdwan, West Bengal. Asian J Transfus Sci.

[17] Giri P, Yadav S, Parhar G (2011). Frequency of ABO and Rhesus blood groups: a study from a rural tertiary care teaching hospital in India. Int J Biol Med Res.

[18] Sidhu S (2003). Distribution of the ABO groups and Rh (D) factor among the scheduled caste population of Punjab. Anthropol.

[19] Shrivastava S, Gahine R, Kapse V (2015). ABO, Rhesus blood group and allele frequency in and around Raipur (Chattisgarh state), India. Int J Cur Res Rev.

[20] Amin MD, Susanto D, Naher L (2015). Distribution pattern of ABO and Rh blood groups and their allelic frequencies among different ethnic groups in Malaysia. Asian Journal of Medical Sciences.

[21] Allawati M, Al-Kalbani L, Al-Balushi T (2021). ABO and Rh (D) phenotypes, allele frequencies and estimated genotypes in Omanis: a retrospective study from Armed Forces Hospital. JMSCR.

[22] Al-Ani L, Mahmood HM, Abdulhaleem N (2020). Genetically determined ABO and (Rh) Rhesus blood groups and their associations with diabetes mellitus. Sys Rev Pharm.

[23] Canizalez-Román A, Campos-Romero A, Castro-Sánchez JA (2018). Blood groups distribution and gene diversity of the ABO and Rh (D) loci in the Mexican population. Biomed Res Int.

[24] Zafar M, Masud S, Malik MM, Latif MH, Noor MS, Asmatullah Asmatullah (2021). Gene frequency distribution of ABO and Rh-D blood group alleles in Multan Division (Punjab), Pakistan. Pure Appl. Biol.

